# Acceptability of a mobile health intervention to enhance HIV care coordination for patients with substance use disorders

**DOI:** 10.1186/s13722-017-0076-y

**Published:** 2017-04-26

**Authors:** Ryan P. Westergaard, Andrew Genz, Kristen Panico, Pamela J. Surkan, Jeanne Keruly, Heidi E. Hutton, Larry W. Chang, Gregory D. Kirk

**Affiliations:** 1 Department of Medicine, University of Wisconsin School of Medicine and Public Health, Madison, WI USA; 2 Department of Epidemiology, Johns Hopkins Bloomberg School of Public Health, Baltimore, MD USA; 3 Department of Population, Family and Reproductive Health, Johns Hopkins Bloomberg School of Public Health, Baltimore, MD USA; 4 Department of International Health, Johns Hopkins Bloomberg School of Public Health, Baltimore, MD USA; 5 Department of Medicine, Johns Hopkins School of Medicine, Baltimore, MD USA; 6 Department of Psychiatry and Behavioral Sciences, Johns Hopkins School of Medicine, Baltimore, MD USA; 7 University of Wisconsin-Madison, 1685 Highland Ave, MFCB 5223, Madison, WI 53705-2281 USA

## Abstract

**Background:**

Persons living with HIV and substance use disorders face barriers to sustained engagement in medical care, leading to suboptimal antiretroviral treatment outcomes. Innovative mobile technology tools such as customizable smartphone applications have the potential to enhance existing care coordination programs, but have not been rigorously studied.

**Methods:**

We developed and implemented a two-component intervention consisting of peer health navigation supported by a smartphone application conducting ecologic momentary assessment (EMA) of barriers to care and medication adherence. Patients with a history of antiretroviral treatment failure and substance use were recruited to participate in the 9-month pilot intervention. Three peer health navigators were trained to provide social and logistical support while participants re-engaged in HIV care. We assessed the acceptability of the intervention components using qualitative analysis of in-depth interviews conducted with study participants and peer navigators.

**Results:**

Of 19 patients enrolled in the study, 17 participated for at least 2 months and 15 completed the entire 9-month study protocol. The acceptability of the peer navigation intervention was rated favorably by all participants interviewed, who felt that peer support was instrumental in helping them re-engage in HIV care. Participants also responded favorably to the smartphone application, but described its usefulness mostly as providing reminders to take medications and attend appointments, rather than as a facilitator of patient navigation.

**Conclusions:**

Peer health navigation and smartphone-based EMA are acceptable approaches to facilitating engagement in HIV care for drug using populations. Future studies to evaluate the efficacy of this approach for improving long-term retention in care and antiretroviral treatment outcomes are warranted.

*ClinicalTrials.gov Identifier* NCT01941108; registered on September 4, 2013

## Introduction

### Background

The development of combination antiretroviral therapy (ART) has had a dramatic impact on the global rate of deaths due to HIV/AIDS. By the end of 2015, an estimated 15.8 million people were receiving ART globally, the large majority of whom reside in low and middle-income countries [1]. When taken consistently as part of a comprehensive package of medical care, ART is highly effective for preventing progression to AIDS and also reduces sexual transmission of HIV, making it a powerful prevention tool.

HIV care is a complex, life-long intervention that requires sustained engagement in care and high-levels of medication adherence in order to confer optimal benefit [2, 3]. These requirements can contribute to health disparities, whereby patients with range of vulnerabilities including mental illnesses, [4] substance use disorders [5] and other challenges [6–8] are less likely to achieve HIV viral suppression. In large cohort studies, people who inject drugs have been demonstrated to have inferior virologic outcomes and higher mortality than other patients receiving ART [9]. To ensure maximal benefit for these populations, social support and/or care coordination strategies are needed to address the specific barriers encountered by people who use drugs when they receive HIV care.

The determinants of poor engagement in HIV care among people who use drugs are heterogeneous and complex. Improving HIV care therefore requires interventions that are individually tailored and flexible. An example of such a strategy is case management, which has been adopted in many HIV care settings as a means to coordinate care for patients with psychosocial needs [10]. Case management, while demonstrated to be effective linking patients to needed services and support, [11] is typically *clinic*-*based*, and therefore may have limited impact for the most highly marginalized patients who have difficulty attending clinic appointments. Patient navigation, an alternative care coordination strategy developed in cancer care settings, provides comparable support using staff who are often para-professional, peer, or lay health workers. Patient navigators are frequently *community*-*based*, and therefore may be able to provide individualized support in the context of patients’ daily lives, rather than primarily inside clinic environments [12].

While more flexible and responsive than traditional case managers, patient navigators typically support a relatively small number of clients, a limitation that may reduce the affordability and scalability of this approach in resource-limited settings. A recent randomized trial showed that patient navigation improved short-term HIV treatment outcomes for hospitalized patients with substance use disorders, but failed to show a significant benefit compared to treatment as usual after 1 year of follow-up [13]. Strategies to improve the efficiency and geographic reach of patient navigators could strengthen their impact on populations at high risk for poor treatment outcomes.

Electronic health (eHealth) and mobile health (mHealth) tools hold promise to move these efforts forward. Mobile phone ownership and use has expanded among people in all income strata [14], including those affected by substance abuse [15] and mental illness, [16] suggesting that mHealth interventions may be increasingly feasible among marginalized groups. Our team’s prior work showed that individuals who report daily drug use are willing to adhere to research protocols involving frequent use of electronic diaries or smartphones for reporting symptoms and responding to surveys [17–19]. Trials of longer-term mHealth interventions have shown benefit for reducing unhealthy alcohol use, [20] and are currently underway to evaluate effectiveness for reducing relapse among individuals with opioid use disorder [21]. Pilot feasibility studies of smartphone applications for self-monitoring of risk behaviors [22] and antiretroviral treatment adherence [23] among HIV-infected substance users have been completed, but larger studies are needed to determine their effectiveness for improving HIV treatment outcomes.

### Study objectives

mPeer2Peer was a two-component intervention that used a smartphone application and patient navigation delivered by peer health workers (“peer navigators”) to support HIV treatment for patients who had been marginally engaged in care. Peer navigators were trained to deliver intensive psychosocial and logistical support for patients with substance use and other barriers to HIV care. Both patients and peer navigators used a smartphone-based mHealth application, which was developed specifically for this study as a means to enhance communication and enable timely, individually tailored support interventions.

This study targeted patients with past or current illicit drug use, whom our prior research has shown to experience frequent lapses in HIV care [24]. Specifically, we aimed to recruit adult patients who were aware of their HIV status, had been linked to HIV care and were prescribed ART, but had not successfully achieved viral suppression. In this paper, we describe the development, implementation and acceptability of the mPeer2Peer intervention, and then discuss implications of the findings from this pilot study to the development of future, larger-scale clinical trials in this population.

## Methods

### Study setting

This study was conducted in Baltimore, Maryland, a city located in the Mid-Atlantic United States with a population of 620,000 and an HIV prevalence of 2377 per 100,000. Over 50% of people diagnosed with HIV in Baltimore report a history of injection drug use, a percentage substantially higher than the general HIV-infected US population [25]. The main clinical site for the study was the Johns Hopkins Moore Clinic, an academic HIV clinical practice serving approximately 2000 patients annually [26]. The mPeer2Peer study protocol was nested within the AIDS Linked to the IntraVenous Experience (ALIVE) study, an ongoing, NIH-funded observational cohort of current and former injection drug users [27].

### Study population

Current and former patients of the Moore Clinic were eligible for mPeer2Peer if they were older than 18, had an HIV viral load greater than 1000 copies/mL, attended no clinic visits with an HIV care provider in the preceding six months, and were willing to attend at least one HIV care visit at the Moore Clinic after enrollment. A formal diagnosis of substance use disorder was an inclusion criterion in the original study protocol, but this requirement was removed mid-study because of difficulty meeting recruitment goals. Exclusion criteria were any medical or psychiatric conditions that would interfere with the participant’s ability to comply with study procedures (e.g., eyesight conditions that would make it difficult to read the smartphone screen) and concurrent participation in other studies focusing on retention in HIV care. Active drug use was not an exclusion criterion.

### Intervention development

**Fig. 1:**
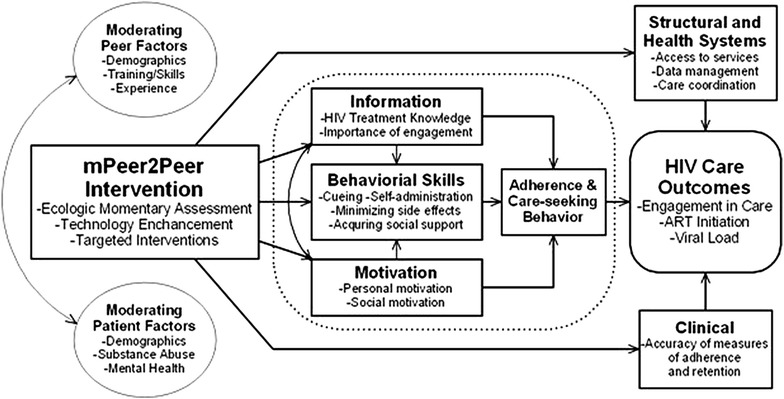
Conceptual framework for mPeer2Peer based on the sIMB model

The mPeer2Peer intervention featured two components, mHealth and peer navigation, which were based on the situated Information, Motivation and Behavioral Skills (sIMB) model of care initiation and maintenance [28]. In this theoretical framework, relevant information, motivation, and behavioral skills interact to determine engagement in care and HIV-related behaviors and outcomes. In the context of this study, the IMB model is influenced by moderating patient and peer factors, as well as structural/health systems and clinical domains, as illustrated in Fig. 1.

The peer navigation intervention was adapted from procedures implemented in a large, multi-site HIV Prevention Trials Network study (HPTN 061), the methods of which have been described previously [29]. The specific duties of the peer navigator were to evaluate patients’ barriers to engagement in HIV care and antiretroviral medication adherence and provide individually tailored support [30]. Three peer navigators were hired who were familiar with the communities in East Baltimore where participants lived, and had experience assisting patients access and utilize health care and social services. All patient navigators received training related to the study procedures, the mHealth application, and basic counseling strategies based on motivational interviewing. Peer navigators were expected to meet face to face with participants in the intervention group at least twice during the first month after enrollment, and interacted with participants in person or via phone calls and text messages on an as needed-basis thereafter.

**Fig. 2:**
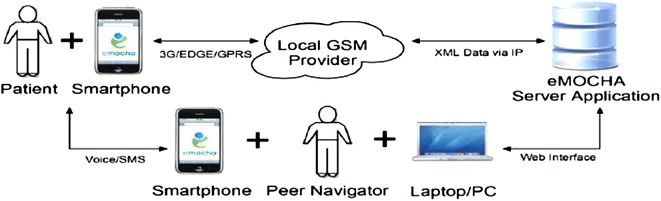
Framework and components the mHealth application

The different components of the mHealth intervention are illustrated in Fig. 2. Intervention group participants and peer navigators each received a smartphone running the Android operating system. The research budget provided for service plans allowing unlimited voice, text and mobile data service throughout the study. Participant data entered at the baseline study encounter and updated regularly by the patient and peer navigator was stored on an encrypted server, which could be accessed and edited via smartphone or using an internet-based application (“dashboard”) by peer navigators. The participant interface of the mHealth application consisted of a password-protected, personalized HIV care summary screen, which displayed updated information about upcoming clinic appointments, recent laboratory results, and contact information for clinic staff on their care team. Participants also had the option of inserting text describing their personal goals or brief motivating statements to be displayed on the summary screen every time the application was launched.

### Ecological momentary assessment

Ecological momentary assessment (EMA) is a method of studying target behaviors and their antecedents by collecting data from research subjects in real time, typically through the use smartphones or other types of electronic diaries. The smartphone application prompted participants to complete brief surveys via the smartphone interface twice daily. One prompt was delivered at a random time of day between 9:00 a.m. and 9:00 p.m. and the second prompt occurred at the end of the day, typically between 9:00 and 10:00 p.m. The precise schedule of prompts could be modified based on participant preferences. The dual purposes of the prompts were (1) to provide reminders about adherence to medications and HIV care visits, and (2) to briefly assess symptoms or behaviors that pose potential threats to ongoing engagement in care, thereby facilitating brief message-based interventions or peer navigator contact. Care engagement surveys were tailored to each participant’s schedule of upcoming appointments using pre-defined algorithms and data fields populated by participants’ responses to prior surveys, and included assessments of medication adherence specific to each participant’s prescribed regimen.

Other variables assessed via daily surveys included craving for drugs or alcohol, use of drugs or alcohol, and mood states. Mood was assessed weekly using the Profile of Mood States-Short Form (POMS-SF) [31], and daily using an abbreviated version of POMS-SF that was limited to symptoms of depression, anxiety, irritability and exhaustion. Under the supervision of the research team and a clinical psychologist, peer navigators reviewed participant-generated data using the web-based interface. Concerning patterns of survey responses related to drug use (e.g. relapsing heroin use after a period of sobriety), or sustained high levels of negative mood states would serve as a trigger for a peer navigator to initiate contact with participants and offer support and linkage to needed services, as appropriate.

### Recruitment and enrollment

A pool of potentially eligible patients was generated using the electronic health records system of the Moore Clinic as part of a clinic-wide initiative to identify and re-engage patients who had lapsed in their HIV care. The mPeer2Peer study inclusion criteria served as the basis of the query, which was conducted using Epic Systems software (Epic Systems Corp., Verona, WI). Clinic staff contacted patients by phone using the contact information contained in their medical records. Patients meeting eligibility criteria who expressed willingness to re-engage in care were invited to meet with the study coordinator in a private off-site research office. After providing demographic and locator data, consenting participants were randomized in equal proportions to receive either (1) usual care or (2) the mPeer2Peer intervention. The experiences of the intervention participants are presented here; a forthcoming manuscript will describe the control group participants and the results from the pilot intervention trial. Participants assigned to the intervention were loaned a smartphone and provided with standardized training on how to use the mHealth application. Afterwards, the study coordinator facilitated a face-to-face meeting between participants and their peer navigator, which typically occurred on the same day as the baseline study visit.

Participants received remuneration in the amount of $25 for completion of the baseline study visit and for each of 3 follow-up study assessments scheduled at 3, 6, and 9 months after enrollment. Additional incentives of $2 per week was offered for responding to 60% or more of their EMA prompts and $5 per week for responding to at least 80% of their EMA prompts. If a participant lost a study smartphone, one replacement device would be dispensed. As a deterrent to device loss, intervention participants received $100 if they returned their original smartphone device at the end of the study and $50 for returning a replacement device. Participants were informed at enrollment that loss of two study devices would result in their dismissal from the study.

### Qualitative evaluation

Acceptability of the intervention was evaluated with one-on-one, in-depth interviews with the first 12 study participants and the 3 peer navigators. Interviews were digitally recorded and professionally transcribed for qualitative analysis by an interdisciplinary research team. Semi-structured interview guides were designed to elicit perceptions about the usefulness of each intervention component, specific needs met by the intervention, and the ease of use of the smartphone application. Interview transcripts were imported into Atlas.ti, a software program for managing and analyzing qualitative data (ATLAS.ti:. Version 7. Statistical Software. (2012) Berlin, Germany: Scientific Software Development). Qualitative data analysis was based on thematic analysis, which aimed to discover themes, as defined as patterns of responses or meaning within the data, through a process of coding, analysis, and thematic mapping [32]. A preliminary codebook was developed based on topics addressed in the interview guides. One of the authors independently coded the transcripts and results were summarized and shared among investigators as a written report after the conclusion of the intervention period.

## Results

Between September 2013 and November 2014, 19 individuals were enrolled and randomly assigned to receive the mPeer2Peer intervention. The baseline characteristics of the intervention recipients are shown in Table 1. The study sample was reflective of the population living with HIV in Baltimore, i.e., predominantly Black, male, and low-income, with a median age of 49. 3 years. Most participants reported they were taking antiretroviral therapy at the time of enrollment, yet all but one had an HIV viral load greater than 1000 copies/mL.

**Table 1:** Baseline characteristics of intervention sample (N = 19)

Characteristic	*N* (%)
Sociodemographic variables	
Median age, years (IQR)	49.3 (45.0–54.6)
African American (%)	17 (89%)
Male (%)	12 (63%)
High school/GED (%)	14 (74%)
Ever married (%)	5 (26%)
Income, yearly < $5000 (%)	8 (42%)
In prison, ever (%)	9 (47%)
Homeless, ever (%)	5 (26%)
Substance use variables*	
Cigarette use (%)	14 (74%)
Alcohol use (%)	8 (42%)
Marijuana use (%)	10 (53%)
Cocaine use (%)	3 (15%)
Heroin use (%)	4 (26%)
Injecting drug use, any (%)	3 (16%)
Clinical variables^†^	
Prescribed ART at enrollment (%)	16 (84%)
Hepatitis C virus seropositive (%)	7 (37%)
Median CD4 (IQR)	171 (95–262)
CD4 < 200 cells/mcL (%)	10 (53%)
Median HIV viral Load (copies/mL) (IQR)	18,938 (3458–103,437)

* Represents self-reported exposure during the 6 months prior to enrollment. *IQR* Intra-quartile range, *GED* graduation equivalence degree, *ART* antiretroviral therapy

^**†**^From health records at time of enrollment

### Study retention and losses to follow-up

Of the 19 patients randomized into the intervention group, 15 (78.9%) were followed for the entire 9-month study period. Two participants were immediately lost to follow-up and had no contact with the study team after the enrollment visit; two others were lost to follow-up after month 2 and 6, respectively. Collectively, these 19 participants contributed 143 person-months of follow-up after enrollment.

### Acceptability of intervention components

Of the 15 participants who completed the study, twelve were interviewed at the end of the study. Interviews elicited uniformly positive assessments of both intervention components, and reported that they found the intervention helpful for supporting their engagement in HIV care. All 12 rated the smartphone application as easy to use or very easy to use. One sixty-two year-old woman reported the system was somewhat difficult in the beginning because she had never used a smartphone before; by the end of the study she reported it was easy to use.

When asked to describe the unmet needs that were addressed by the mHealth application, the most common benefit reported by participants was that it provided reminders to take medications and attend appointments. Participants who described their daily lives as disorganized or chaotic indicated that prompts to complete EMA surveys helped them focus on their HIV care at times when they felt distracted by other sources of stress, as described by one participant as follows:

“It was helping me stay a grip with things that was going on in my life, which was drugs, and it was a reminder because it would ask me, ‘Did you use?’ And I was able to say, ‘No, no,’ you know, so it did help me, especially with my appointments and what my goals was it did help…. I’m gonna miss that now. I just hope and pray that everything goes still smooth. I haven’t missed an appointment since then.” (Participant, 58 year-old woman)

Several participants expressed disappointment that they would no longer have access to the emocha application after their participation in the study ended. Several asked for assistance to find and download a freely available smartphone application that would allow them a way to continue to receive reminders.

“They told me about the app and it was a free download and I downloaded it on my regular phone. ‘Cause I liked it so much. It just it doesn’t feed into your database…. ‘cause it’s a whole different app itself. But I did set up a reminder for it.” (Participant, 48 year-old man)

Participants expressed the belief that the intervention would be beneficial for other patients facing similar challenges, and even felt that their experience in the study would empower them to provide support for other patients.

“By me helping myself I will help others, because I had some associates that was dealing with stuff by not being compliant, and I had this phone. It was like, ‘How can I get in this program? Because I need something.’ Because they go through a lot throughout the day and you forget, like I forgot things today. So having that phone, that reminder is so good. It was so good.” (Participant, 37 year-old man)

The peer navigation intervention component was also very well-received by participants, who strongly believed that the social support they received facilitated their engagement in HIV care. As described by participants, the nature of the support provided by peer navigators appeared multi-faceted. Some participants valued the presence of a peer who is available to listen and provide encouragement in in a non-judgmental way, while at other times the peer navigator served as an advocate for their health care in a more direct way.

“She’s very smart, she works with you not against you, you know, and that’s what I like about her…they’re the kind of people I like to have in my life.” (Participant, 55 year old man)

“[My Peer Navigator] is one of those people that you just know she’s in your corner. [She] listened. She didn’t judge. She didn’t nag. She just listened to what you have to say.” (Participant, 48 year old man)

“I had to go in the hospital and she was right there. She walked it through everything that I needed. Thank God she was there because she helped the hospital process move along. So they wouldn’t forget me in the emergency room where—I remember one time I was in there and they kept me on a bedpan for almost an hour and a half. And when [peer navigator] called and she said, “How’s it going,” and I told her, and before I knew it somebody was in that room. She’s really helpful, and I appreciate it and I love her. That’s my girl.” (Participant, 58 year-old woman)

From the perspective of peer navigators, both intervention components appeared to provide important support for patients who had historically struggled to remain engaged in HIV care. The most frequently mentioned benefits perceived by peer navigators were their roles as a peer educator and as a source of support that mitigated against patients’ experiences of depression and social isolation:

“For some of them, some of their barriers were depression. So they just weren’t self-motivated to do things. And just having me call them once a week to check in, just to have a conversation, and to show them other outlets of where they can go and talk to people was helpful too.” (Peer-navigator, 36 year-old woman)

All 3 peer navigators interviewed agreed that EMA prompts served as medication reminders, which were important facilitators of improved antiretroviral adherence.

“The navigation worked well. For most of them, the phone worked well. A lot of them more than anything appreciated the reminders. The appointment reminders and the take their medication [reminders] ‘cause some of ‘em just forget to take their meds.” (Peer Navigator, 68 year-old woman)

Not clearly reflected in the interviews was the hoped-for impression that the mHealth application was *integrated* into the peer navigation intervention. Specifically, we did not observe that the data collected using EMA informed the peer navigators’ activity on a regular basis. One of the peer navigators acknowledged how the content of the EMA surveys addressed more upstream determinants of medication adherence and overall engagement in HIV care, but perceived that the surveys were beneficial in their own right, rather than as a tool to improve her ability to support patients:

“The ones that suffered with mental health issues, depression mostly, they really liked the mood questions. It helped them gauge how they were feeling and it was a way of getting it out, you know what I mean, it was a way of expressing themselves, it made them feel a little bit better, even if it was temporary relief. (Peer Navigator, 36 year-old woman)

One of the three peer navigators described an instance in which data collected through an EMA survey informed her interactions with a participant in a meaningful way. The peer navigator, who periodically reviewed the EMA survey results of her clients, discovered that one had reported using drugs at a time she had previously believed he was in recovery. This provided an impetus to contact the participant by phone and discuss the issues surrounding his relapse, and to ensure the patient was continuing to take his medications and would attend his next clinic appointment. The other two peer navigators reported that they found it difficult to find time to log into the emocha server application to review participant data in real time, and did not find it to be a useful resource for their interactions with participants. Rather, they viewed the mHealth component as a distinct intervention that offered benefits that were different from peer navigation

“I know they liked the phone they did find it useful… they also appreciated more the human contact. Knowing somebody cared. Knowing that if I got an issue I can call this person up.

## Discussion

In this pilot study, we implemented a novel mHealth-supported patient navigation intervention designed to improve HIV treatment utilization and outcomes among people with substance use disorders. Our findings suggest that people living with HIV in urban communities characterized by high levels of poverty and substance abuse find both intervention components acceptable and responsive to the types of barriers encountered when accessing HIV care.

Electronic health (eHealth) and mobile health (mHealth) strategies are new and potentially powerful approaches to coordinating complex, longitudinal medical care. Technology can empower patients, with direct delivery of individualized motivation, education, and support. Internet-based tools can facilitate communication among patients, providers, and supportive peers. Wireless technologies remove the barriers of time and distance between patients and providers. These advantages are especially important for substance-using patients and other populations that are hard to reach and difficult to keep engaged in care. Technology-based interventions may therefore serve as valuable adjuncts to case management and patient navigation programs, which deliver high-impact support but are expensive and challenging to disseminate on a large scale.

Our study adds to the growing literature suggesting that patient navigation and mHealth interventions are acceptable, client-centered strategies to improve engagement in health care. Patient navigation has been implemented among groups with shared experiences and challenges, such as HIV-positive women of color, [33], adults undergoing complex evaluations for cancer, [34] and persons leaving correctional facilities [35]. The high-level of acceptability of the smartphone intervention in this sample of older adults (i.e., half were older than 50) is notable. Social isolation is an important risk factor for adverse health outcomes among aging individuals living with HIV [36]. Prior research has suggested the benefits of mHealth and eHealth for improving care for chronic conditions are mediated by enhancement of social support [37, 38]. If true, this provides reason for optimism that the now ubiquitous opportunities or online social networking can be leveraged to fill unmet social support needs among people who are aging and have complex health needs.

Most participants endorsed the peer navigation intervention as a source of needed support, and believed that having a phone provided by the study facilitated their interactions with the peer navigators. Whether a standard mobile phone without the mHealth application would have served this purpose equally well is not clear. Some participants enjoyed receiving the EMA surveys, and even found the periodic monitoring of mood and drug cravings to be a source of support in and of itself. We did not find strong evidence that peer navigators reviewed and acted upon the EMA data provided by participants on a regular basis, which was one of rationales for developing this two-component intervention. This may reflect several limitations of our approach. For example, the internet-based dashboard did not display participants’ EMA data in a way that was easy for peer navigators to visualize and interpret. While peer navigators had dedicated office space and internet access, the majority of their work was done outside of the clinic, and reviewing EMA data was not incorporated into their workflow on a consistent basis. Increasing the nature or intensity of peer navigator training and oversight may improve this aspect of the intervention, and should be explored in future projects of this type.

Other potential limitations of this study deserve mention. Based on the query of electronic health records, there were a number of patients who had lapsed in their HIV care who could not be contacted or were not willing to participate in the study. It is therefore possible that the participants in this study represent a subset of out-of-care patients who are more responsive to interventions such as mPeer2Peer, and that this approach may be less acceptable to patients with more intractable barriers to engagement in HIV care. Participants received modest financial incentives for responding to EMA surveys. While this was felt to be necessary to ensure the pilot study generated useful data, this approach may have biased participants’ perceptions of the acceptability of the intervention. Whether adherence to the intervention protocol would be similar in routine practice settings as we observed in this research study is not known. A criticism of the mHealth field in general is that findings from small pilot studies have not been translated to larger scale projects that can make an impact on a population level [39].

## Conclusions

This study demonstrates that interventions using patient navigation bundled with an mHealth application are feasible and acceptable to patients with substance use disorders who have failed to remain consistently engaged in HIV care in the past. Challenges highlighted by our research include the potential difficulty of integrating technology-based tools into the workflow of lay treatment supporters such as peer navigators. Additional research to inform implementation strategies, and ultimately to determine the efficacy of these interventions for improving engagement in HIV care and viral suppression, is warranted.

## Abbreviations

HIV: human immunodeficiency virus
EMA: ecologic momentary assessment
ART: antiretroviral therapy
AIDS: acquired immunodeficiency syndrome
ALIVE: AIDS Linked to the IntraVenous Experience study
NIH: National Institutes of Health
sIMB: situated information, motivation, and behavioral skills model
GPS: global positioning systems
HPTN: HIV Prevention Trials Network
POMS-SF: profile of mood states-short form
eHealth: electronic health
mHealth: mobile health
